# Effects of school meals with weekly fish servings on vitamin D status in Danish children: secondary outcomes from the OPUS (Optimal well-being, development and health for Danish children through a healthy New Nordic Diet) School Meal Study

**DOI:** 10.1017/jns.2015.15

**Published:** 2015-07-17

**Authors:** Rikke A. Petersen, Camilla T. Damsgaard, Stine-Mathilde Dalskov, Louise B. Sørensen, Mads Fiil Hjorth, Rikke Andersen, Inge Tetens, Henrik Krarup, Christian Ritz, Arne Astrup, Kim F. Michaelsen, Christian Mølgaard

**Affiliations:** 1 Department of Nutrition, Exercise and Sports, Faculty of Science, University of Copenhagen, Denmark, Rolighedsvej 26, DK-1958 Frederiksberg C, Denmark; 2 Division of Nutrition, The National Food Institute, Technical University of Denmark, Denmark, Mørkhøj Bygade 19, DK-2860 Søborg, Denmark; 3 Department of Clinical Biochemistry, Section of Molecular Diagnostics, Aalborg University Hospital, Hobrovej 18–22, DK-9000 Aalborg, Denmark

**Keywords:** Vitamin D, Nutrition, Children, School meals, 25(OH)D, 25-hydroxyvitamin D, BA, bone area, BMC, bone mineral content, BMD, bone mineral density, DXA, dual-energy X-ray absorptiometry, IGF-1, insulin-like growth factor-1, OC, osteocalcin, OPUS, Optimal well-being, development and health for Danish children through a healthy New Nordic Diet, PTH, parathyroid hormone

## Abstract

Children's vitamin D intake and status can be optimised to meet recommendations. We investigated if nutritionally balanced school meals with weekly fish servings affected serum 25-hydroxyvitamin D (25(OH)D) and markers related to bone in 8- to 11-year-old Danish children. We conducted an explorative secondary outcome analysis on data from 784 children from the OPUS School Meal Study, a cluster-randomised cross-over trial where children received school meals for 3 months and habitual lunch for 3 months. At baseline, and at the end of each dietary period, 25(OH)D, parathyroid hormone (PTH), osteocalcin (OC), insulin-like growth factor-1 (IGF-1), bone mineral content (BMC), bone area (BA), bone mineral density (BMD), dietary intake and physical activity were assessed. School meals increased vitamin D intake by 0·9 (95 % CI 0·7, 1·1) μg/d. No consistent effects were found on 25(OH)D, BMC, BA, BMD, IGF-1 or OC. However, season-modified effects were observed with 25(OH)D, i.e. children completing the school meal period in January/February had higher 25(OH)D status (5·5 (95 % CI 1·8, 9·2) nmol/l; *P* = 0·004) than children completing the control period in these months. A similar tendency was indicated in November/December (4·1 (95 % CI –0·12, 8·3) nmol/l; *P* = 0·057). However, the effect was opposite in March/April (–4·0 (95 % CI –7·0, –0·9) nmol/l; *P* = 0·010), and no difference was found in May/June (*P* = 0·214). Unexpectedly, the school meals slightly increased PTH (0·18 (95 % CI 0·07, 0·29) pmol/l) compared with habitual lunch. Small increases in dietary vitamin D might hold potential to mitigate the winter nadir in Danish children's 25(OH)D status while higher increases appear necessary to affect status throughout the year. More trials on effects of vitamin D intake from natural foods are needed.

Data suggest that a considerable number of healthy European children and adolescents have circulating vitamin D concentrations below the currently recommended level of 50 nmol/l^(^^1^^)^, and ways to increase childhood status are continuously sought for. The primary source of vitamin D in humans is the synthesis that occurs upon sun exposure of the skin^(^^2^^)^. Also, evidence of an effect of vitamin D-containing supplements and fortified foods on vitamin D status appears evident^(^^3^^,^^4^^)^. Although secondary to these, dietary vitamin D is likewise believed to be an important contributor to circulating vitamin D levels, and it has persistently been shown that European children's vitamin D intake can be optimised to meet recommendations^(^^5^^,^^6^^)^. Foods such as meat and milk have been found to contribute to the intake of dietary vitamin D^(^^7^^)^, but only a few foods, primarily fatty fish, are naturally rich in vitamin D^(^^8^^)^. There is a general lack of studies investigating the effect of diet on vitamin D status in children, and questions remain as to whether it is possible to affect vitamin D status through realistic intakes of natural food sources and what proportions such intakes must have to have an effect.

Although current evidence of links between vitamin D status and bone health in healthy children is inconsistent^(^^9^^,^^10^^)^, sufficient childhood vitamin D status is thought to be crucial to facilitate optimal Ca supply, bone formation and bone health, potentially decreasing the risk of fractures and osteoporosis in later life^(^^11^^)^. Indeed, skeletal Ca accretion increases vastly during childhood growth^(^^12^^)^. Such increased Ca requirements may result in reduced serum Ca which leads to stimulation of parathyroid hormone (PTH). PTH enhances the hydroxylation of 25-hydroxyvitamin D (25(OH)D) in the kidneys, generating the active vitamin D compound, 1,25-dihydroxyvitamin D (1,25(OH)_2_D). Actions of 1,25(OH)_2_D include increased intestinal Ca absorption, and, in combination with PTH, potential mobilisation of Ca from bones^(^^13^^)^. In this way, vitamin D and PTH work to maintain Ca homeostasis in the blood at the potential expense of Ca in bone. Vitamin D status, measured as 25(OH)D, is found inversely correlated with PTH in elderly individuals^(^^14^^)^, and studies have generally also observed this association in children, although not as systematically and consistently^(^^15^^,^^16^^)^. Though not fully elucidated, other informative biomarkers with relation to vitamin D and bone include osteocalcin (OC), a protein synthesised by osteoblasts and marker of bone formation^(^^17^^)^, and insulin-like growth factor-1 (IGF-1), a growth hormone also thought to be involved in bone regulation and development^(^^18^^)^.

We report secondary and explorative outcomes from a large school meal intervention trial conducted in Danish children. It has previously been reported that this intervention moderately but highly significantly increases the children's intake of dietary vitamin D^(^^19^^)^. Therefore the aim of the present study was to exploratively investigate whether any effects of the intervention could be found on the children's: (1) serum 25(OH)D; and (2) serum PTH, bone mineral density (BMD), bone mineral content (BMC), bone area (BA), plasma OC and IGF-1. Due to negligible vitamin D synthesis from sun exposure during winter at northern latitudes^(^^20^^,^^21^^)^, we worked with an *a priori* hypothesis that the intervention's impact on vitamin D status could vary according to seasonal months.

## Methods

### Study design and subjects

**Fig. 1.**
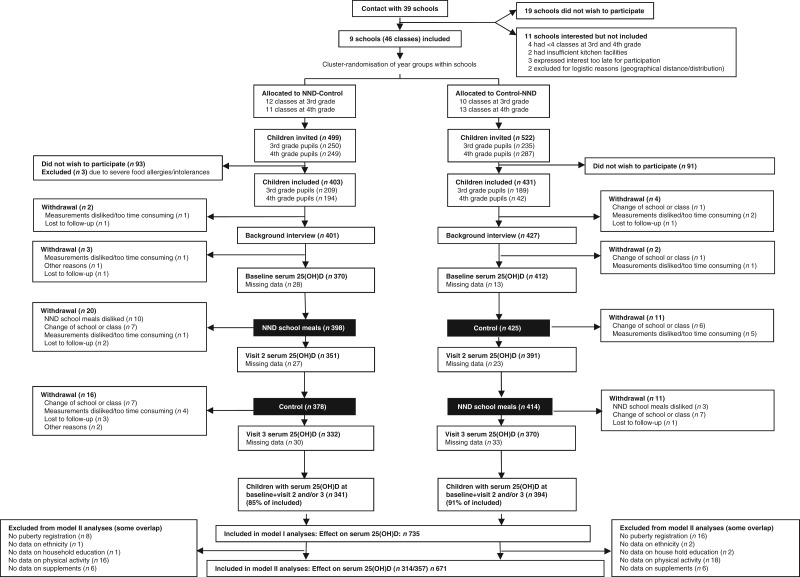
Flowchart of the study, illustrating the flow from recruitment of the OPUS (Optimal well-being, development and health for Danish children through a healthy New Nordic Diet; NND) schools to the measurements of the main outcome in the present study, i.e. serum 25-hydroxyvitamin D (25(OH)D) at baseline, visit 2 and visit 3.

The present study is based on data from the OPUS (Optimal well-being, development and health for Danish children through a healthy New Nordic Diet) School Meal Study, a large cluster-randomised controlled cross-over trial that ran from August 2011 to June 2012. The overall aim of the OPUS School Meal Study was to assess the impact of providing school meals based on the New Nordic Diet to third- and fourth-grade children and the primary outcomes were a continuous metabolic score and a measure of the children's concentration performance. The design and methods of the study have been described in detail elsewhere^(^^22^^)^. Briefly, third- and fourth-grade children at nine Danish public schools received school meals for 3 months preceded or succeeded by 3 months with intake of their habitual packed lunch. In Denmark packed lunches often contain open-faced rye-bread sandwiches with cold cuts but can take a wide variety of forms. The sequence of intervention and control periods was assigned randomly at each school in clusters corresponding to grade, i.e. at each school either all third or fourth graders were assigned to start with the intervention period while the opposite grade started with the control period. Initial contact was established with thirty-nine schools whereof nine were included (Fig. 1). Inclusion criteria for the schools were: (a) location in the eastern part of Denmark (Zealand and Lolland-Falster); (b) a total of four or more classes at third and fourth grade; (c) suitable kitchen facilities available for food production during school days; and (d) a high motivation for participation as determined by the study team. Written information about the study was sent to the families of all third- and fourth-grade children at the nine participating schools and the families were invited to an information meeting. Exclusion criteria for the invited children were: (a) diseases or conditions that might obstruct the measurements or put a child at risk by eating the intervention school meals (for example, severe food allergies); or (b) concomitant participation in other scientific studies that involved radiation or blood samples. Consent for participation was obtained from 834 children, corresponding to 82 % of the 1021 invited children. A successive study start of the schools meant that data collection ran throughout the whole school year of 2011–2012. Clinical measurements were performed at baseline, at the end of the first dietary period (visit 2) and at the end of the second dietary period (visit 3). Baseline ran from 30 August 2011 to 28 November 2011, visit 2 ran from 29 November 2011 to 12 March 2012, while visit 3 ran from 13 March 2012 to 25 June 2012.

### Ethics

The present study was conducted according to the guidelines laid down in the Declaration of Helsinki and all procedures involving human subjects were approved by the Committees on Biomedical Research Ethics for the Capital Region of Denmark (H-1-2010-124). The trial was registered in the database www.clinicaltrials.gov (no. NCT 01457794). Written informed consent was obtained from all custody holders of each child.

### Intervention

The school meals consisted of a hot lunch meal and two daily snacks served at the school. Lunch and snacks aimed to meet 40–45 % of the children's daily energy intake based on the energy requirement of an 11-year-old boy^(^^23^^,^^24^^)^. All school meals were prepared locally at each school by trained chefs and had emphasis on foods such as fish, whole grains, coarse vegetables, nuts and berries as defined in the New Nordic Diet guidelines^(^^25^^,^^26^^)^. Fish was served twice per week during the intervention period; once per week as the main dish, and once per week as part of a lunch buffet. Menus and types of fish varied according to seasons. Meals were served *ad libitum* with guidance to achieve a nutritionally balanced intake, and the 15 min usually set aside for eating during the lunch break in Danish schools were increased to 20–25 min during the school meal period. The effects of the school meals on the children's dietary intake are reported in detail elsewhere^(^^19^^)^. Here, it was reported that the children's fish intake was 48 % higher in the school meal period compared with the control period, while that of vitamin D was 42 % higher^(^^19^^)^.

### Sociodemographic characteristics and puberty stage

Information about demographic and socio-economic characteristics was collected at baseline by an in-depth 2 h personal interview with each child and his/her parents. Parental education level was defined as the highest education level attained in the household, i.e. lower secondary education (≤10 years), upper secondary education, vocational education, short higher education, bachelor's degree or equivalent, or ≥ master's degree (≥17 years). Based on birth countries of parents and grandparents, children were categorised as ‘immigrants/descendants’ if all grandparents and one or both parents were born outside Denmark. Based on breast development in girls and pubic hair development in boys (Tanner stages) pubertal status was self-evaluated by the child with assistance by the parents^(^^27^^)^. Registrations were dichotomised into a variable indicating whether the child had entered puberty (≥Tanner stage 2) (yes/no).

### Dietary intake, physical activity and outdoor school time

Registrations of dietary intake, and physical activity, were performed before each visit, i.e. baseline, visit 2 and visit 3, respectively. With assistance from their parents, children recorded their daily intake of food and beverages for seven consecutive days using Web-based Dietary Assessment Software for Children (Web-DASC) developed specifically for this age group in the OPUS School Meal Pilot Study^(^^28^^)^. Intake data were processed by a General Intake Estimation System (GIES), originally developed for the Danish National Survey of Diet and Physical Activity (DANSDA) 2003–2008 (Division of Nutrition, The National Food Institute, Technical University of Denmark, Denmark)^(^^7^^)^. Energy intake (EI) relative to estimated BMR was evaluated following the equations by Henry^(^^29^^)^, and energy under- (EI/BMR ≤ 1·05) and over-reporters (EI/BMR ≥ 2·28)^(^^30^^)^ were excluded from the dietary variables, as were children who registered their diet for less than 4 d. Dietary intake did not include intake from supplements as these were registered separately during the 7 d dietary recordings. These data allowed for a summarisation of the intake of vitamin D-containing supplements, including multivitamins, on a yes/no basis for each day of dietary registration. Vitamin D supplement intake was applied without excluding children who recorded for less than 4 d and without excluding energy over- and under-reporters. A variable signifying the intake of vitamin D-containing supplements in percentage (days with supplements intake/total number of days of dietary recording) was applied in the present study.

To measure physical activity, the children were asked to wear an accelerometer (ActiGraph GT3X or GT3X+ Tri-Axis Accelerometer Monitor) in an elastic belt tightly at the right hip for the same 7 d and eight nights as the dietary recordings, and to remove it only during water activities, i.e. showering or swimming. Analyses of the physical activity data are described in detail elsewhere^(^^31^^)^. The derived variable was time per d in moderate-to-vigorous physical activity (≥2296 counts per min)^(^^32^^)^. From a questionnaire to the principal at each school, information was obtained on whether the children were obligated to go outdoors during all recesses, and how many min per week the classes approximately spent outdoors during school days on walking between classrooms and other school facilities.

### Anthropometry, dual-energy X-ray absorptiometry scans and ethnicity

Anthropometric measurements and dual-energy X-ray absorptiometry (DXA) scans were conducted at each school at baseline, at visit 2, and at visit 3. Anthropometry measurements were conducted during morning hours after the child had been fasting since midnight, had emptied the bladder, and was wearing light clothing only. Height was measured in centimetres to the nearest one decimal using a transportable stadiometer (CMS Weighing Equipment Ltd). Height was derived as the mean of three consecutive measurements with the child in standing position, holding his head in the Frankfurter plane. Body weight was measured in kilograms to the nearest one decimal using a digital scale (Tanita BWB 800 S). BMI was calculated as (body weight (kg))/(height^2^ (m)). Waist circumference was measured in centimetres to the nearest millimetre using a non-elastic measuring tape at the level of the umbilicus and the mean of three consecutive measurements was used. Total body less head (TBLH) BMC (measured in grams of hydroxyapatite), bone size expressed as anterior–posterior TBLH BA (measured in cm^2^), and BMD (calculated as BMC (g)/BA (cm^2^)), were determined by DXA scan using a Lunar Prodigy ProTM (GM Healthcare) with EnCoreTM software version 13.5. DXA quality controls were conducted daily and weekly using an AP Spine phantom (LUNAR 17466). DXA scans were conducted throughout the day, and most children had therefore had a standardised breakfast before they were scanned. At each scan, the investigator recorded the child's ethnicity according to the terminology of the DXA software, i.e. Caucasian, Asian, African, Latin or other. Children from Turkey, the Middle East, Pakistan and India were defined as Caucasians. Due to few recordings in certain categories, recordings were dichotomised into a variable indicating whether the child was Caucasian (yes/no).

### Blood sampling and analyses

Blood sampling was conducted at the same occasions as anthropometric measurements and DXA scans. Local anaesthetic patches were offered to the children to reduce discomfort, and blood was sampled by venepuncture after an overnight fast. Serum was separated by centrifuging collected blood at 2500 ***g*** at 4 °C for 10 min after 30 min at room temperature. Serum and plasma were hereafter stored at –80 °C for later analyses. For each analysis, all samples were ran on the same device with the same reagent lot, all samples from each child were analysed on the same day, and all samples from each school were analysed in one assay. As a measure of vitamin D status, 25(OH)D concentration (25(OH)D_2_ + D_3_; DTOT25) was assessed in serum by automatic chemiluminescence immunoassay (CLIA) technology on a DiaSorin LIAISON (DiaSorin AB) at the Department of Clinical Biochemistry, Aalborg University Hospital, Aalborg, Denmark, a laboratory partaking in the vitamin D external quality assessment scheme (DEQAS)^(^^33^^)^. Before analysis, serum was thawed at room temperature for 30 min before centrifugation at 4000 rpm for 3 min. Concentrations are presented in nmol/l (1 nmol/l = 0·4006 ng/ml). Two 25(OH)D samples were under the detection limit of 10 nmol/l at visit 2; these were applied in analyses as half of the detection limit (5 nmol/l). Serum intact PTH concentrations were determined using the CLIA technique on an ADVIA Centaur XP (Siemens Healthcare). Two PTH samples, one at baseline, and one at visit 3, were below the detection limit of 0·265 pmol/l; these were applied as half of the detection limit (0·135 pmol/l) in the analyses. One baseline PTH sample of 109 pmol/l (25(OH)D at 89·1 nmol/l) was excluded from data as an extreme outlier. Plasma IGF-1 and OC were determined using the CLIA technique on an Immulite 1000 (Siemens Healthcare Diagnostics). Concentrations are presented in ng/ml (1 ng/ml = 1 µg/l). One child had OC concentrations above the detection limit of 100 ng/ml at all visits, while two additional OC samples were above the detection limit at visit 3. These samples were applied in analyses as the detection limit (100 ng/ml). The inter- and intra-assay CV were 5·4 and 7·6 % (25(OH)D); 7·4 and 7·9 % (intact PTH); 2·4 and 2·9 % (IGF-1); and 5·9 and 4·1 % (OC).

### Statistics

Characteristics of the study population are based on the 784 children who contributed data on all or either of the 25(OH)D, BMC, BA and BMD outcomes, i.e. children who had a baseline measurement and a measurement from visit 2 and/or visit 3 of any of these outcomes. To confirm previous findings and warrant credibility of our first aim, investigating the potential effect on vitamin D status, we first performed an evaluation of the effect of the school meals on the dietary vitamin D intake in this particular study sample. An available-case analysis was used. The linear mixed model analysis of recorded vitamin D intake (excluding supplements) included the intervention (school meals/control), visit, order of school meal and control periods, baseline outcome concentration, sex and baseline age as fixed-effects explanatory variables. School, class and child were included as random effects to account for the hierarchical study design. To account for the fact that the school meals changed according to season we additionally did this analysis with the adjustment of the season variable presented below, and we examined whether a school meal and season interaction effect was found on vitamin D intake.

Second, the effect of the school meals on 25(OH)D, BMC, BA, BMD, PTH, OC and IGF-1 was likewise evaluated using linear mixed models considering both a simple model I, and an additionally adjusted model II. Model I included the same effects as the model with dietary vitamin D intake, as stated above. Model II additionally included a season variable, indicating the months in which visit 2 and 3 fell, i.e. November/December, January/February, March/April, or May/June. These months-intervals were included to account for seasonal fluctuations in the children's serum 25(OH)D throughout the study. November/December samplings (*n* 254) and January/February samplings (*n* 423) included visit 2 samples only, whereas March/April samplings included a mix of visit 2 (*n* 107) and 3 (*n* 361) samples, and May/June (*n* 396) included only visit 3 samples. In addition, the following time-dependent fixed effects were included in model II: entered puberty, i.e. Tanner stage ≥ 2 (yes/no), vitamin D-containing supplement intake (days with supplement intake/total number of days of dietary recording at baseline, visit 2, and 3), min/d of moderate-to-vigorous physical activity (baseline, visit 2, and 3), body weight (baseline, visit 2, and 3), height (baseline, visit 2, and 3), waist circumference (baseline, visit 2, and 3), obligated to spend school day recesses outdoors (yes/no), and min/week of outdoors walking between classrooms during school days. Also, Caucasian (yes/no), immigrant/descendant background (yes/no) and parental education level were included as fixed effects in model II to account for the school- and year-grade-level randomisation of the study. In models I and II BMC was additionally size-adjusted for BA. Third, with serum 25(OH)D as outcome we extended model II to examine the *a priori* hypothesised interaction effect between the school meals and months of visit, and due to the potential interrelation between 25(OH)D and PTH, this effect was also evaluated with PTH as outcome.

Assumptions underlying all statistical models were assessed graphically using residual and normal probability plots, and outcomes were logarithm transformed if appropriate. Significance was established at *P* < 0·05. Data analyses were performed by STATA (Stata Statistical Software Release 12.1; StataCorp LP).

## Results

### Study population and characteristics

Baseline characteristics are presented in Table 1. The majority of children in the study were normal weight, non-immigrant/descendants and Caucasian. No sex differences were observed in height, weight and weight status at baseline but boys were older, spent more time on moderate-to-vigorous physical activity, had higher mean serum 25(OH)D concentration, higher BMC, BA and BMD, higher energy intake, and higher intake of dietary vitamin D and Ca than girls. More girls than boys had entered puberty (≥ Tanner stage 2), and girls had higher concentrations of PTH, plasma OC and plasma IGF-1 compared with boys (*P* < 0·05). The sex differences in vitamin D and Ca intake disappeared when calculated per MJ (data not shown).

**Table 1.** Characteristics of the study population (784 children) at baseline who contributed data to at least one of 25-hydroxyvitamin D (25(OH)D), bone mineral content (BMC), bone area (BA) or bone mineral density (BMD) (Mean values and standard deviations, medians and interquartile ranges (IQR), or percentages)

	Girls (*n* 378)		Boys (*n* 406)		
	Mean or median	sd or IQR	Mean or median	sd or IQR	P*
Sex (%)	48		52		
Age (years)	9·9	0·6	10·0	0·6	0·001
Puberty stage: 1/2/3/4/5 (%)†	53·4/36·6/9·7/0·3/0		76·4/19·5/3·8/0/0·3		<0·001
Height (cm)	142·1	7·0	142·8	7·1	0·16
Weight (kg)	33·6	29·7–38·2	34·3	30·2–39·7	0·18
Weight status: underweight/normal weight/overweight/obese (%)‡	11·9/74·9/11·6/1·6		8·6/77·8/11·1/2·5		0·48
Waist circumference (cm)	62·7	58·9–68·3	62·5	59·3–68·2	0·93
Immigrants/descendants (%)	10·6		12·6		0·39
Caucasians (%)	94·4		94·3		0·94
Parental education: A/B/C/D/E/F (%)§	6·1/4·0/31·8/9·5/29·2/19·4		5·4/2·7/30·7/9·4/28·5/23·3		0·77
Moderate-to-vigorous physical activity (min/d)||	38	26–49	57	39–74	<0·001
Obligated outdoors recesses (% yes)	64·8		61·1		0·44
Outdoors walking at school (min/week)	5	0–10	5	0–10	0·46
Serum 25(OH)D (nmol/l)	58·5	18·0	63·0	19·1	0·001
Serum PTH (pmol/l)	3·2	2·4–4·2	3·1	2·2–4·0	0·02
Plasma OC (ng/ml)	30·1	23·4–38·2	24·6	19·9–31·4	<0·001
Plasma IGF-1 (ng/ml)	212	178–269	178	139–209	<0·001
TBLH BMC (g)	927·5	208·2	969·0	199·4	0·005
TBLH BA (cm^2^)	1176·6	179·8	1209·2	173·2	0·01
TBLH BMD (g/cm^2^)	0·78	0·06	0·79	0·06	0·001
Supplement intake: 0/1–2/3–6/7 d (%)¶	47·6/12·7/24·9/14·8		51·7/9·9/24·1/14·3		0·5
Energy intake (kJ/d)	7204	1182	8304	1410	<0·001
Fish intake (g/d)	11	0·4–27	12	0–30	0·57
Fatty fish intake (g/d)	2	0–14	0	0–12	0·21
Dietary vitamin D intake (μg/d)	1·7	1·3–2·5	2·0	1·5–2·9	0·002
Dietary Ca intake (mg/d)	840	682–1006	953	800–1182	<0·001

PTH, parathyroid hormone; OC, osteocalcin; IGF-1, insulin-like growth factor 1; TBLH, total body less head.

* Sex differences were tested using two-sample *t* tests (unequal variance) or Wilcoxon–Mann–Whitney tests (non-normally distributed variables) for continuous variables, and Pearson's χ^2^ tests or Fisher's exact tests for categorical variables.

† Tanner stages as validated by Morris & Udry^(^^27^^)^.

‡ Based on age- and sex-specific cut-offs defined to pass through BMI of 18·5, 25 and 30 kg/m^2^ at age 18 years, as described by Cole *et al.*^(^^48^^,^^49^^)^.

§ A = ≤ lower secondary education; B = higher secondary education; C = vocational education; D = short higher education; E = bachelor's degree or equivalent; F = ≥ master's degree.

|| Moderate-to-vigorous physical activity ≥2 296 counts per min, measured by accelerometer^(^^32^^)^.

¶ Recorded intake of vitamin D-containing supplements, including multivitamins, during the 7 d baseline dietary recording.

### Effect of the school meals on registered dietary vitamin D intake

Exclusion of dietary recordings for less than 4 d as well as under- and over-reporters resulted in 683, 617 and 548 valid recordings at baseline, visit 2 and 3, respectively, corresponding to 87, 79 and 70 % of the 784 children. Children recorded a 0·9 (95 % CI 0·7, 1·1) μg/d (*P* < 0·001) higher intake of vitamin D in the school meal period than in the control period. Compared with the median dietary vitamin D intake of 1·9 (25th–75th percentile 1·4–2·9) μg/d at baseline this corresponded to a 31–64 % increase in vitamin D consumption. An additional adjustment for season did not change the results on vitamin D intake, and a potential school meal and season interaction effect on the vitamin D intake was not found (*P* value for New Nordic Diet and season interaction effect = 0·22).

### Effect of the school meals on 25-hydroxyvitamin D, bone mineral content, bone area, bone mineral density, parathyroid hormone, osteocalcin and insulin-like growth factor-1

Overall, no effect of the school meals was observed on serum 25(OH)D, BMC, BA, BMD, plasma IGF-1 or OC in the simple or adjusted model compared with the habitual lunch (Table 2). However, the school meals were found to affect PTH as they resulted in a 0·18 (95 % CI 0·07, 0·29) pmol/l (*P* = 0·001) increased serum PTH concentration in model I and a 0·23 (95 % CI 0·12, 0·34) pmol/l (*P* < 0·001) increased PTH concentration in model II.

**Table 2.** Crude values according to visit, and the effects of the intervention school meals on 25-hydroxyvitamin D (25(OH)D), bone mineral content (BMC), bone area (BA), bone mineral density (BMD), parathyroid hormone (PTH), osteocalcin (OC) and insulin-like growth factor 1 (IGF-1) (Mean values and standard deviations, medians and interquartile ranges (IQR), or β values and 95 % confidence intervals)

	Crude values*						Effect†				Effect†			
	Baseline (September–November)		Visit 2 (November–March)		Visit 3 (March–June)		Model I‡				Model II‡§			
Outcome	Mean or median	sd or IQR	Mean or median	sd or IQR	Mean or median	sd or IQR	β_school meals_	95 % CI	*n*	*P*	β_school meals_	95 % CI	*n*	*P*
25(OH)D (nmol/l)	60·8	18·7	46·1	17·8	48·6	16·7	0·23	−0·99, 1·45||	735	0·71	0·38	–0·70, 1·46||	659	0·493
PTH (pmol/l)	3·1	2·3–4·1	3·6	2·6–4·8	3·6	2·6–4·9	0·18	0·07, 0·29||	689	0·001	0·23	0·12, 0·34||	614	<0·001
TBLH BMC (g)	949·0	204·6	989·1	218·1	1046·0	234·1	0·08	−0·91, 1·07||¶	780	0·87¶	0·22	−0·78, 1·22||¶	703	0·664¶
TBLH BA (cm^2^)	1193·5	177·0	1232·0	187·9	1279·6	197·4	0·45	−1·81, 2·72||	780	0·70	0·07	−2·25, 2·40||	703	0·950
TBLH BMD (g/cm^2^)	0·79	0·06	0·80	0·06	0·81	0·06	0·00	−0·00, 0·00||	780	0·95	0·00	−0·00, 0·00||	703	0·828
OC (ng/ml)	27·5	21·2–33·9	28·4	22·5–34·3	31·8	25·6–39·3	−0·21	−0·71, 0·28||	732	0·40	−0·02	−0·55, 0·51||	654	0·951
IGF-1 (ng/ml)	193	159–234	198	160–241	202	165–254	−1·17	−3·75, 1·41	732	0·38	−1·35	−4·0, 1·29	654	0·317

TBLH, total body less head.

* Includes children with data from baseline, visit 2 and/or visit 3 on the respective outcome.

† Analyses performed by linear mixed models with school, class and subject as random effects.

‡ Adjusted for visit, order of intervention *v.* control period, baseline value of respective outcome, baseline age and sex.

§ Additionally adjusted for months of visit (November/December, January/February, March/April, May/June), entered puberty (yes/no), intake of vitamin D-containing supplements (days with supplement intake/total number of days of dietary recording), moderate-to-vigorous physical activity (min/d), height, weight, waist circumference, obligated to spend school day recesses outdoors (yes/no), and outdoors walking between classrooms during school days (min/week), Caucasian (yes/no), immigrant/descendant background (yes/no) and parental education level.

|| Serum 25(OH)D, BMC, BA, BMD, serum PTH and plasma OC back-transformed from logarithm transformation in model I and II analyses.

¶ Additionally adjusted for BA.

### Effects of the school meals on 25-hydroxyvitamin D were modified by months of visit

**Fig. 2.**
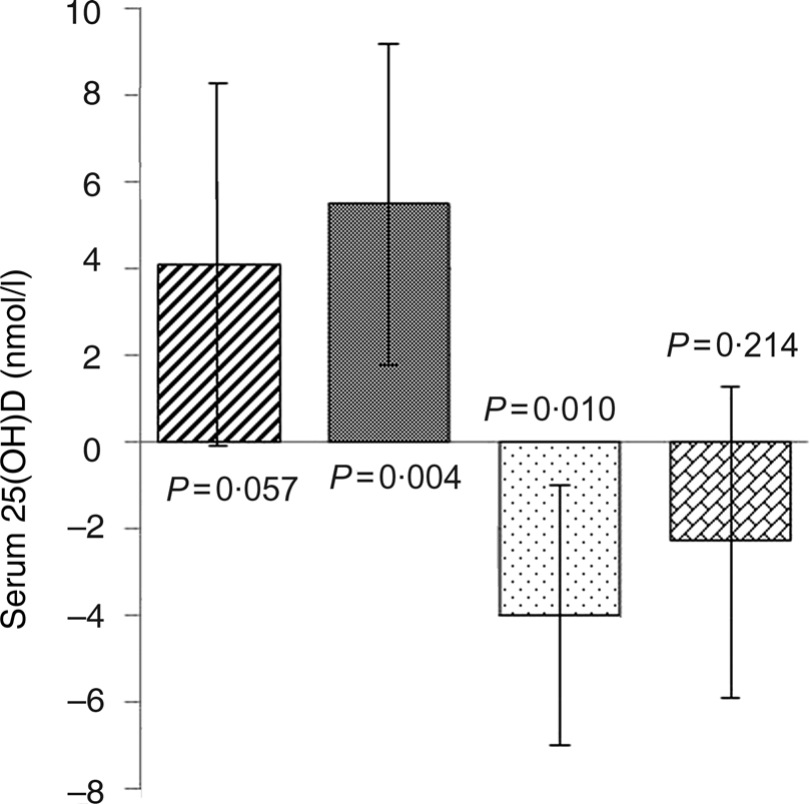
Effect of intervention school meals on serum 25-hydroxyvitamin D (25(OH)D) modified by months of visit in the intervention group compared with the control group, with 0 being the concentration in the control group at the given time: November/December (///); January/February (▓); March/April (░); May/June (▒). Values are estimated effects, with 95 % confidence intervals represented by vertical bars. Adjusted for visit, order of intervention *v.* control period, baseline value of respective outcome, baseline age, sex, months of visit (November/December, January/February, March/April, May/June), entered puberty (yes/no), intake of vitamin D-containing supplements (days with supplement intake/total number of days of dietary recording), moderate-to-vigorous physical activity (min/d), height, weight, waist circumference, obligated to spend school day recesses outdoors (yes/no), and outdoors walking between classrooms during school days (min/week), Caucasian (yes/no), immigrant/descendant background (yes/no), and parental education level, and included the interaction term between school meals and months of visit. Back-transformed from a logarithm transformation.

A significant interaction effect between school meals and months of visit was observed for serum 25(OH)D in model II (*P* = 0·012). This season-modified effect meant that children who completed the school meal period in January/February had a 5·5 (95 % CI 1·8, 9·2) nmol/l (*P* = 0·004) higher 25(OH)D concentration than the children who completed the control period in these months. Indication of a similar tendency was observed in November/December (4·1 (95 % CI –0·12, 8·3) nmol/l; *P* = 0·057). The effect was opposite in March/April, i.e. children who completed the school meal period in March/April had a 4·0 (95 % CI –7·0, –0·9) nmol/l (*P* = 0·010) lower 25(OH)D status compared with the children who completed the control period in these months. No difference was found between children completing the two dietary periods in May/June (*P* = 0·214) (model II). These trends are illustrated in Fig. 2. An interaction effect between school meals and months of visit was not observed with PTH (*P* = 0·29).

## Discussion

The daily vitamin D intake of the children included in this secondary outcome study increased by 31–64 % during the *ad libitum* school meal period compared with the control period. This is a substantial effect considering the low intake continuously reported in European children^(^^5^^,^^6^^)^ and considering the scarce amount of food sources that are naturally rich in vitamin D. As fish intake was reported to increase by 48 % during the school meal period compared with a median intake of 9 g/d in the control period^(^^19^^)^, the increased intake of vitamin D was presumably achieved primarily through the weekly fish servings. Despite these results, no consistent effect of the school meals was found on the children's circulating vitamin D concentrations. Although an effect on vitamin D status of orally ingested vitamin D is evident in the literature, these findings are based on randomised controlled trials with supplementation or fortification^(^^3^^,^^4^^,^^34^^)^ and achieved by much larger intake doses than the present study. Indeed, it must be considered that the habitual intake of fatty fish (median of 0 g/d in the control period) and vitamin D in general (median of 1·9 µg/d in the control period) was minimal in the OPUS School Meal Study^(^^19^^)^. And the reported increases of 48 and 42 %, respectively, still leave low intakes as compared with the currently recommended vitamin D intake of 10 µg/d in Danish children^(^^35^^)^. Consequently, one explanation for the lack of effect on the children's vitamin D status is likely to be that the intake increase was too small to affect serum status.

The small but highly significant school meal effect on PTH was unexpected. Overall, PTH concentrations did appear to have an opposite pattern from serum 25(OH)D, i.e. generally higher at visits 2 and 3 than at baseline (Table 2). However, it is noteworthy that the effect on PTH does not appear to be explained merely by a potential inverse association with the children's serum 25(OH)D concentrations. Indeed, no consistent negative effect of the school meals was found on serum 25(OH)D, and, contrarily to 25(OH)D, no interaction effect between school meals and months of visit was found with PTH. It is interesting to consider that PTH has been recognised to increase as a reflection of adolescent growth^(^^11^^,^^15^^,^^36^^)^, and, although highly hypothetical, a potential rationale for the unexpected effect on PTH might be a speculated growth-related stimulation triggered by the school meals. It is noteworthy that the dietary Ca intake (mean 845–872 mg/d) was generally adequate in the OPUS School Meal Study, and that Ca intake was unaffected by the school meals^(^^19^^)^. However, the school meals did increase the intake of, for example, protein, vitamin C, P, Se, iodine, Fe, Mg and K^(^^19^^)^, and although beneficial effects of the school meals have been reported on blood pressure, plasma TAG and insulin resistance, they also affected waist circumference by a slight 0·5 cm increase compared with the control period^(^^37^^)^. This underlines that the present explorative results must be interpreted with consideration to the fact that the study affected several aspects of the children's dietary intake and health.

The significant interaction effect between school meals and months of visit partially supported our *a priori* hypothesis of a varying effect of the school meals according to seasonal months. In line with some cross-sectional observations^(^^38^^,^^39^^)^, the season-modified effect of the school meals on vitamin D status indicates to a certain degree that dietary vitamin D is increasingly influential under conditions of negligible cutaneous vitamin D synthesis such as winter months at northern latitudes. Indeed, a beneficial effect of the school meals on 25(OH)D status was present only in the latest winter months of January/February while a borderline positive effect (*P* = 0·057) was found in November/December, and no difference was found in May/June when sunshine is abundant. Overall, the 25(OD)D level dropped from a mean of 60·8 nmol/l at baseline (September–November) to 46·1 nmol/l at visit 2 (November–March) (Table 2). Hence, the 5·5 nmol/l higher serum 25(OH)D concentration found in January/February with the school meals may be of clinical importance to mitigate the winter nadir well known to occur in children's vitamin D status at northern latitudes^(^^40^^–^^43^^)^. The school meal effect found in January/February corresponded to a 6·1 nmol/l increase in circulating vitamin D per 1 µg increased daily intake of vitamin D from natural food sources. No studies are available for suitable magnitude comparisons, as previous studies have been conducted with higher doses and from supplements and fortification. Yet, for proportion measure, one study on vitamin D supplementation in Danish and Finish adolescent girls found 25(OH)D to increase by 2·43 nmol/l per 1 µg higher total vitamin D intake (diet and supplemental) in the end of winter^(^^44^^)^, and an observational study in elderly British long-stay female hospital patients with no access to sunlight showed an increase in serum 25(OH)D of 5·2 nmol/l per 1 µg/d increase in vitamin D_3_ intake^(^^45^^)^. Still, the total vitamin D intake varied between subjects and studies as did the sources of vitamin D. Interestingly, it has recently been suggested that the input of vitamin D from dietary sources is substantially larger than traditionally predicted due to contributions from foods originally unaccounted for as conventional vitamin D food sources^(^^46^^)^. Also, the bioavailability and type of ingested vitamin D might differ between sources^(^^46^^,^^47^^)^ and complicate effect comparisons between supplementation and natural food studies.

However, the lower 25(OH)D concentrations observed in children receiving school meals in March/April compared with children receiving habitual lunch was unexpected and make the season-modified effect of the school meals inconclusive. We do not expect the varying fish type servings to explain this dissimilar effect. Indeed, we did not find the school meal intervention effect on vitamin D to be modified by season. Also, winter and spring menus differed only in one of three fish types and both contained salmon, which was the fish serving with the highest content of vitamin D^(^^8^^)^. We do speculate, however, that the extra time required for eating the school meals compared with the habitual lunch may have resulted in less outdoors recess time during the school meal period compared with the control period. During winter months this might be unimportant while it might be highly relevant in March/April as sunlight starts to regain position as the primary vitamin D source.

The present study was a secondary outcome investigation and a direct measurement of the children's daily time outdoors and extent of sunny holidays was not available. This would, however, have strengthened the analyses. The inclusion of the interaction term between school meals and months of visit in the statistical analyses can be discussed as it shifts investigations from within-subject to between-subject comparisons with the risk of the observed effects being random differences between the children sampled at each specific months-interval. Still, the *a priori* hypothesis on which these analyses were based was supported by strong evidence of seasonal fluctuations in the children's vitamin D status. Also, it strengthens these analyses that more than 250 children were sampled in each months-interval and that we were able to adjust for a vast variety of potential confounders in model II. The consecutive study start of the schools was a challenge to the analyses, yet due to the comprehensive data collection and school meal servings at each school a simultaneous study start for all schools was not logistically possible. We acknowledge that a longer intervention period is probably needed to fully assess potential effects on children's bone health. Also, it is a limitation to the study that the specific doses of the vitamin D-containing supplements were not available. Still, we were able to apply a repeated supplements variable that contained information on the frequency of supplement intake relative to the amount of days with dietary recordings in three registration periods throughout the study in each child. One may argue that the *ad libitum* servings of the intervention meals was a limitation to the study since the actual intake was less controlled, and it must be acknowledged that the intake of, for example, fish probably varied between children. On the other hand, the design highly resembles a real-life setting in a large and highly representative sample of Danish school children and it provided power to detect even small effects^(^^22^^)^ in a field that has not been explored until now. Also, although the achieved increase in vitamin D intake is likely to have been too small to have an overall effect on status it is an important finding that children's vitamin D intake can increase through realistic servings of natural foods. In conclusion, servings of nutritionally balanced school meals positively affected children's dietary intake of vitamin D, yet the increased intake of 0·9 µg/d appears insufficient to consistently affect vitamin D status in healthy children. However, season-modified effects indicate that intake increases of such small magnitude might hold potential to mitigate the winter nadir in vitamin D status at northern latitudes. However, these results were inconsistent and appear to suggest the importance of future school meal studies to assure that recess and time spent outdoors are not compromised by the intervention. More randomised controlled trials on the effect of vitamin D intake from natural food sources on markers of vitamin D and bone are needed. These should have longer and parallel study periods and achieve higher vitamin D intake than that of the present study. This appears highly feasible, as the present study exploratively reported from a trial with other primary aims. Although small, the unexpected effect of the school meals on PTH also raises questions for further investigations.
